# External validation of a COPD prediction model using population-based primary care data: a nested case-control study

**DOI:** 10.1038/srep44702

**Published:** 2017-03-17

**Authors:** Bright I Nwaru, Colin R Simpson, Aziz Sheikh, Daniel Kotz

**Affiliations:** 1 Asthma UK Centre for Applied Research, Centre for Medical Informatics, Usher Institute of Population Health Sciences, The University of Edinburgh, UK; 2 School of Health Sciences, University of Tampere, Finland; 3 Department of Family Medicine, CAPHRI School for Public Health and Primary Care, Maastricht University Medical Centre, Maastricht, The Netherlands; 4 Institute of General Practice, Addiction Research and Clinical Epidemiology Unit, Medical Faculty of the Heinrich-Heine-University Düsseldorf, Düsseldorf, Germany

## Abstract

Emerging models for predicting risk of chronic obstructive pulmonary disease (COPD) require external validation in order to assess their clinical value. We validated a previous model for predicting new onset COPD in a different database. We randomly drew 38,597 case-control pairs (total *N* = 77,194) of individuals aged ≥35 years and matched for sex, age, and general practice from the United Kingdom Clinical Practice Research Datalink database. We assessed accuracy of the model to discriminate between COPD cases and non-cases by calculating area under the receiver operator characteristic (ROC_AUC_) for the prediction scores. Analogous to the development model, ever smoking (OR 6.70; 95%CI 6.41–6.99), prior asthma (OR 6.43; 95%CI 5.85–7.07), and higher socioeconomic deprivation (OR 2.90; 95%CI 2.72–3.09 for highest vs. lowest quintile) increased the risk of COPD. The validated prediction scores ranged from 0–5.71 (ROC_AUC_ 0.66; 95%CI 0.65–0.66) for males and 0–5.95 (ROC_AUC_ 0.71; 95%CI 0.70–0.71) for females. We have confirmed that smoking, prior asthma, and socioeconomic deprivation are key risk factors for new onset COPD. Our model seems externally valid at identifying patients at risk of developing COPD. An impact assessment now needs to be undertaken to assess whether this prediction model can be applied in clinical care settings.

The burden of chronic obstructive pulmonary disease (COPD) has been rising, now representing one of the leading causes of morbidity and mortality worldwide^1^. Whilst estimates of disease burden have primarily come from developed countries, the prevalence appears to be rising in developing countries as well, and the resultant mortality is projected to rise by 30% in the next decade^2^. In the United States (US), over 12 million adults have COPD, representing the third leading cause of death^3^; it is the second most common cause of emergency hospitalisation in the United Kingdom (UK)^4^,^5^,^6^.

Whilst some studies have evaluated algorithms to identify individuals with established COPD^7^,^8^,^9^, the rate of undiagnosed disease remains high^10^, occurring in one out of eight people over the age of 35 years^6^. There is a paucity of studies that have developed hands-on tools that enable early identification of individuals^9^ at-risk of future COPD. Our ability to construct models that will enhance identifying individuals at risk well before disease onset will provide the opportunity for developing key strategies for prevention^11^. Using the Primary Care Clinical Informatics Unit (PCCIU) general practice (GP) database, we developed and internally validated the first risk prediction model for early detection of incident COPD, which simultaneously took into account a range of known risk factors, including smoking, age, sex, prior asthma, and socio-economic status^11^. A more recent study utilised the UK Clinical Practice Research Datalink (CPRD) database to similarly develop and validate a COPD prediction model, deriving comparable predictive values as our previous study^12^. However, it is important, prior to using these risk scoring systems in clinical practice that they are externally validated in entirely different datasets of comparable populations.

In the current study, we therefore aimed to externally validate our previously developed prediction model (developed using the PCCUI GP database) using a different database (i.e. the CPRD GP database). The current work is the first to externally validate a COPD prediction algorithm for early detection of individuals at-risk of future COPD in an entirely different database but comparable population.

## Results

### Background characteristics of the study population

Overall, majority of the patients had smoked at some point, but the proportion of smokers was higher in COPD cases (*n* = 33,269; 86%) than in controls (*n* = 19,908; 52%), regardless of whether the CPRD smoking codes or the PCCIU codes were used (Table 1). The proportion of those with prior asthma was up to *n* = 13,161, 17% (cases *n* = 10,210, 27%; controls *n* = 2,951, 7%). A higher proportion of cases was more deprived than controls as measured by the Index of Multiple Deprivation (IMD) quintile (Table 1).

### Associations between risk factors and COPD

In unadjusted and adjusted (i.e. simultaneous adjustment for all factors) models, ever smoking was associated with a seven-fold increased odds of COPD (Table 2). Prior asthma was associated with an increased risk of COPD: adjusted OR for prior asthma was 5.04 (95% CI 4.77–5.33). Compared to the least deprived IMD quintile, those in the more deprived IMD quintiles were increasingly at higher risk of COPD (Table 2). The estimates for smoking and prior asthma were similar in magnitude and direction when either of the codes from CRPD and PCCIU data were used in the analyses (data not shown).

### Validation of COPD prognostic index

**Figure 1:**
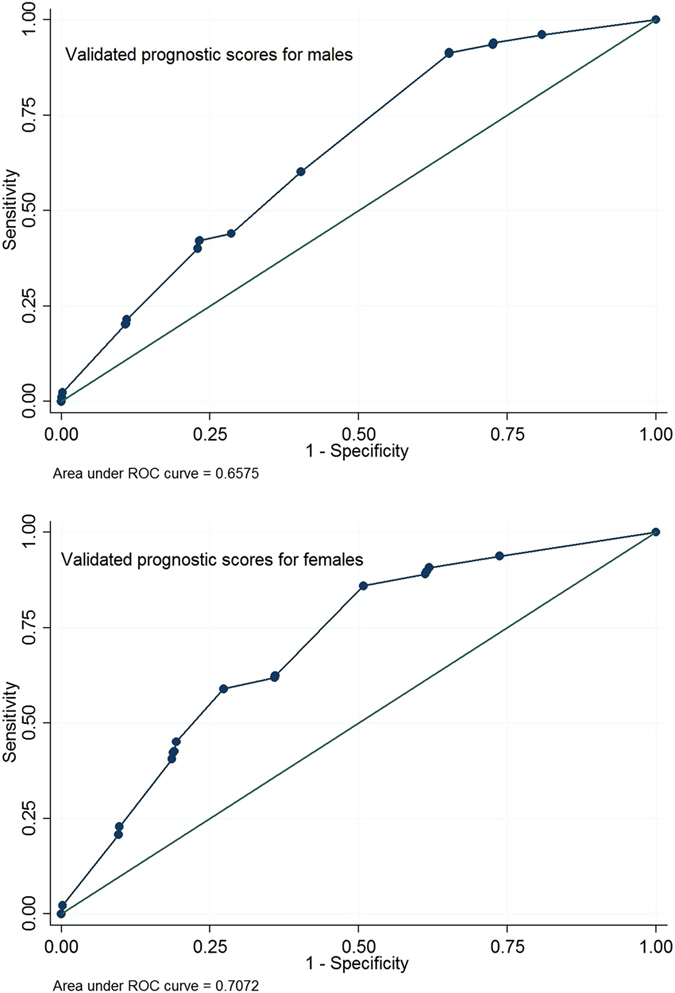
ROC curves for the validated prognostic scores, for males (top) and females (bottom): the prediction model developed using the PCCIU data was applied to the CPRD data.

Table 3 presents the validated decile prognostic scores when the scores derived from the development models based on the PCCIU data were applied to the CPRD data. The scores were derived for males and females separately and ranged from 0 (lowest) to 5.71 (highest) for males and 0 to 5.95 for females. Some deciles did not have corresponding prognostic scores, an indication that the scores were not normally distributed. The accuracy of the validated prediction model in discriminating between COPD and non-COPD patients was ROC_AUC_ = 0.66 (95% CI 0.65–0.66) for males and ROC_AUC_ = 0.71 (95% CI 0.70–0.71) for females (Table 3). The ROC curves for the validated scores are shown in Fig. 1 and the sensitivity and specificity values for the various cut points on the prognostic scores are shown in Supplementary File 4.

### COPD prognostic scores derived from the CPRD data

Table 4 presents the prognostic scores derived solely from the CPRD data. The scores ranged from 0 to 4.37. The corresponding ROC_AUC_ for assessing the accuracy of the model was 0.74 (95% CI 0.73–0.74). The ROC curves are shown in Figure S1 of Supplementary File 5. The prognostic scores were similar when either of the smoking and prior asthma codes from CRPD and PCCIU data were used in the analyses (data not shown).

## Discussion

In this first ever external validation exercise of a COPD prediction model using the CPRD database, we have found similar prediction estimates as those derived from the PCCIU database derived based model, both in the magnitude and direction of effect. Whilst the prognostic scores based on the development model ranged from 0 to 7.50, similarly for males and females, the scores from the current validation study ranged from 0 to 5.71 for males and 0 to 5.95 for females. Similarly, whilst we observed some small differences in the associations between smoking, prior asthma, and deprivation quintiles and the risk of COPD, these were largely in the same direction of impact as expected. As expected, the accuracy of the models as measured by the ROC_AUC_ were somewhat lower in this validation study (males 0.66, females 0.71) compared to the development study (males 0.83, females 0.85); nonetheless, these estimates still have the potential to be very useful in clinical practice.

The CPRD database is a well-characterised population-based primary care database and is one of the best validated large primary care research databases in the world^13^,^14^. With sufficient sample size and power, we have – for the first time –validated a COPD prediction score in a different dataset, obtaining similar risk factors and analogous accuracy estimates to discriminate COPD cases from non-cases compared to the measures derived from the prediction development study^11^. In comparison to the prediction development study using the PCCIU database^11^, the coding for smoking and asthma was more complete in the current study using the CPRD data. No differences were however seen when either of the CPRD- and PCCIU-derived codes were used.

A limitation of this study is that it was based on a matched case-control design but nested in a longitudinal population cohort (with estimation of the conditional odds ratios of the influence of the risk factors on the development of COPD), whereas the prediction development study was based on a follow-up cohort study (with estimation of the hazard ratios of the influence of the risk factors on a 10-year risk of COPD). Nevertheless, the estimates of associations between the various risk factors and the development of COPD were both in the direction and magnitude comparable between the validation and prediction development studies. Although the measures of accuracy (using the ROC_AUC_) of the prediction model in discriminating between COPD cases and controls were slightly lower in the current study compared to the prediction development study, the differences were as expected, given previous evidence in this respect, i.e. accuracy measures of prediction models more often are seen to be lower in an externally validated dataset compared to estimates derived from the prediction development dataset^15^. This work has therefore served to confirm the importance of undertaking external validation studies. Further limitation of our work is that, spirometry measures, which are the gold standard for diagnosing COPD, are not routinely recorded in the GP databases, hence we could not utilise them in this study. Similarly, pack-years of smoking, a desired exposure indicator to assess the causal impact of smoking, is not routinely collected in GP databases, hence we did not consider it in this study.

As the first validation of a risk score for predicting the development of COPD in a different external database, there is no applicable previous study to compare the results. Overall, the risk estimates for the studied risk factors (smoking, prior asthma, and socioeconomic status) were comparable to the estimates from the prediction model development study. Whilst a recent study using CPRD undertook an external validation of the prediction scores, the validation work was done within the same database: the authors split the original data into two (development and validation samples) datasets, using one dataset to develop the prediction scores and the second dataset comprising of 20 CPRD general practices to validate the scores^12^. External validation of prediction models aims to assess the generalisability of the derived model in an appropriate similar patient population, but in a different context; this work therefore needs to be undertaken in a new dataset^15^,^16^,^17^.

In comparison to the recent study using CPRD database to develop and validate a prediction model^12^, the risk estimates with regards to smoking and asthma, although in the same direction as observed in the current study, somewhat differed in magnitude, possibly as different definitions were used between the two studies. In the current study we defined smoking as “ever smoking” while the previous study differentiated between former and current smoking^12^. Another difference between the current study and the previous CPRD study was that variables included in the final model differed, which may explain the differences observed: whilst the final model in the current study included smoking status, prior asthma, and IMD, the previous study included smoking status, prior asthma, salbutamol prescription, and lower respiratory tract infections. Further difference relates to the time coverage of participants’ enrolment into the two studies: the current study included participants between 1992 and 2012, whereas the previous study covered between 2000 and 2006. The UK Quality and Outcomes Framework (QOF) for COPD started in 2004, at which point the coding of COPD improved^5^,^6^. However, since both studies contained data both prior to and after the start of the QOF, we believe the time coverage of studies may not have substantially influenced the observed variations in results.

Other previous studies from the US^7^,^8^ and Denmark^9^ developed prediction algorithms aimed at identifying COPD patients with already established disease. They were also based on secondary care data, such as administrative claims data, outpatient pharmacy data, and hospital admissions data, hence contrast the current study, which used population-based primary care to validate prediction scores aimed at identifying at-risk individuals before the onset of disease.

As the incidence of COPD and mortality continues to rise globally, our ability to detect cases before they manifest is crucial^6^,^11^,^12^. The current COPD prediction model, now externally validated in a different dataset, but comparable population, provides a convincing opportunity for evaluating its usefulness and applicability in clinical care settings for identifying individuals at high risk of developing the disease^18^. The current validation confirms the importance of smoking history, prior asthma, and socioeconomic status^19^, which in combination provide a composite prediction score to accurately identify individuals at different risk categories (based on the our prediction model, low category had risk scores ≤6, medium risk score of 7, and high risk score 8–10) of developing COPD^11^. The current study is the first to externally validate a COPD prediction score. Whilst emerging COPD prediction models from other contexts also need to be externally validated in different datasets, we believe that the risk scores derived from the current external validation exercise can now be assessed whether it can applied in clinical settings with a concurrent impact assessment of its performance.

In conclusion, using a large internationally respected database, we have – for the first time – validated a COPD prediction model for identifying at-risk individuals prior to the onset of disease in a different database but comparable population, with acceptable accuracy at discriminating between COPD cases and non-COPD cases. Key predictors of onset of COPD include smoking, prior asthma, and socioeconomic status. Consideration now needs to be made, with a concurrent impact assessment on performance, on whether the validated risk score is useful and can be readily applied in clinical practice for identifying those at risk of developing COPD.

## Methods

### Study population

The CPRD database is a validated computerised, anonymised and longitudinal primary care database, considered by many as the gold standard of routine clinical research data (www.cprd.com)^13^,^14^. It is jointly funded by the UK National Health Service (NHS) and the Medicines and Healthcare products Regulatory Agency (MHRA). Data are linkable to other healthcare and social care data sources, and are regularly used to conduct both observational and interventional research in the UK. Presently the database comprises around 14 million patients derived from 660 primary care practices across the UK^13^,^14^. We received access to CPRD under licence from the Medical Research Council (MRC). The CPRD Group has obtained ethical approval from a Multi-centre Research Ethics Committee for all purely observational research using CPRD database. The protocol of the current study was approved by the Independent Scientific Advisory Committee of CPRD (protocol number 10_084 R). All the study methods were performed in accordance with the relevant guidelines and regulations and in accordance with best scientific practices.

We extracted a random sample from the CPRD of COPD cases and controls aged ≥35 years: index cases and their corresponding controls were matched at a ratio of 1:1 on GP practice, sex, and year of birth (within two years). Cases comprised of individuals with first recorded COPD diagnosis and who were followed for at least five years prior to the index date (i.e., date of drawing the sample). Identification of cases and controls and definition of other study variables were based on the Read Clinical Classification System, a standard coding system produced for clinicians in primary care and which is used for most primary care electronic patient records in the UK (a complete list of Read codes used for this study is given in Supplementary File 1). A control must have been registered at the same practice at the time of the index date of the corresponding case and should have had at least five years of follow-up in the same practice prior to the index date of the case. The index date for the controls was the date of the recording of COPD for the corresponding matched case.

In total, we sampled 38,597 case-control pairs (total *N* = 77,194). This was the maximum number of allowable patients extracted under the Medical Research Council license. Of these, 188 (84 [0.22%] cases and 104 controls [0.27%]) had missing data for IMD, hence were excluded from analyses. The IMD is the UK government’s measure to assess household’s socio-economic status based on the level of deprivation of an area (http://census.ukdataservice.ac.uk/get-data/related/deprivation). Two controls without ID numbers and their corresponding cases were further excluded, resulting in a total of 77,002 (38,511 cases and 38,491 controls) as the final sample for analyses.

### Assessment and definition of risk factors

From CPRD, we extracted the same variables used in the PCCIU data to develop the prediction model (i.e., smoking, prior asthma, and IMD); the current study sample was matched for age and sex. Smoking status was categorised into “never smoker” (i.e., patients recorded as “non-smoker” at any time and no coding as “smoker” or “ex-smoker” at any other time) or “ever smoker” (i.e., patients recorded as “smoker” or “ex-smoker” at any time); “never smoker” was the reference category. Prior asthma was categorised as “no” or “yes”; “no” was the reference category. As the study was nested within a longitudinal cohort, the timing of occurrence of asthma was determined and we ensured that only asthma cases occurring prior to COPD were defined as positive. IMD was categorised into quintiles (quintile 1 as least deprived and quintile 5 as most deprived); quintile 1 was the reference category.

We observed some differences in coding of smoking and asthma between the CPRD and PCCIU data: in comparison to the codes in PCCIU, CPRD comprised of four extra codes for smoking status and 15 extra codes for asthma (see supplementary File 1). This resulted in about 0.5% extra ever smokers and about 12% extra prior asthma cases by using CPRD codes compared with PCCIU codes. The Read code, H33.00 (Asthma), contributed to most (85%) of the extra asthma cases in CPRD. Whilst we used CPRD-derived codes throughout the validation exercise, we also separately analysed PCCIU-derived codes to assess whether there were any differences between the two codes in terms of the direction and magnitude of the prediction scores.

### Statistical analysis

The Stata codes for data preparation and analyses are presented in Supplementary Files 2 and 3, respectively. For descriptive analysis we calculated the frequencies of participants’ background characteristics by cases and controls. Assessment of the associations between the risk factors and the risk of COPD was performed by calculating the unadjusted and adjusted risk estimates using conditional logistic regression. These estimates are reported as odds ratio (OR) and accompanied by their 95% confidence intervals (95% CI). It should be noted that while the development model was based on a longitudinal data (hence modelling using Cox regression), the current validation work is based on a matched case-control study (hence the calculation using conditional logistic regression).

A two-part approach was used to undertake the external validation of the prediction model. First, the risk scores derived from the prediction model (based on coefficients across categories of the variables included in the fully adjusted Cox regression model)^11^ were applied to the corresponding categories of each predictor in the validation model, thus deriving the prognostic scores for each individual in the validation model of the CPRD data; this was done separately for males and females. The resulting risk scores were then divided into 10 risk categories (deciles). The approach is described in Table 5.

For example, a 60-year-old male ever smoker, with IMD level 3 and no previous history of asthma would yield a prognostic index (PI) of 5.0 (risk category 9 = high risk of future COPD); a 40-year-old female never smoker, with IMD 3 and a previous history of asthma would yield a PI of 2.2 (risk category 3 = low risk of future COPD).

In the second part of the validation model, the prognostic scores for each individual was derived solely based on the CPRD data using the method applied in the model development: i.e., calculation of the risk scores as the sum of the regression coefficients across the different categories of the variables in the fully adjusted conditional logistic regression model. Due to the CPRD validation dataset being matched for sex and age, the scores were calculated for males and females combined and not separated by sex. The resultant risk scores were then divided into deciles. Illustratively, given the beta regression coefficients for the categories of the different factors as **β**, calculation of the risk scores was given as follows:


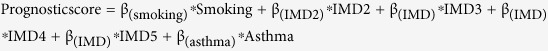


Where β_(smoking)_*Smoking is the coefficient for a smoker vs never smoker as the reference; β_(IMD2)_*IMD2 is the coefficient for an individual in the second quintile of IMD to β_(IMD)_*IMD5 for an individual in the highest quintile of IMD vs firs quintile as the reference; and β_(asthma)_*Asthma for an individual with asthma vs non-asthma as the reference.

For each validation model, the accuracy of the prognostic scores in discriminating between COPD and non-COPD patients was estimated by calculating the area under the receiver operator characteristic curve (ROC_AUC_) for all values of the scores.

## Additional Information

**How to cite this article:** Nwaru, B. I. *et al*. External validation of a COPD prediction model using population-based primary care data: a nested case-control study. *Sci. Rep.* **7**, 44702; doi: 10.1038/srep44702 (2017).

**Publisher's note:** Springer Nature remains neutral with regard to jurisdictional claims in published maps and institutional affiliations.

## Supplementary Material

Supplementary Information

## Figures and Tables

**Table 1:** Participants’ baseline characteristics by COPD cases and controls.

Characteristic	All *N* = 77,002 *n* (%)	COPD Cases *n* = 38,511 *n* (%)	Controls *n* = 38,491 *n* (%)
Smoking status			
Never smoker	23,825 (30.9)	5,242 (13.6)	18,583 (48.3)
Ever smoker	53,177 (69.1)	33,269 (86.4)	19,908 (51.7)
Previous asthma prior to diagnosis of COPD			
No	63,841 (82.9)	28,301 (73.5)	35,540 (92.3)
Yes	13,161 (17.1)	10,210 (26.5)	2,951 (7.7)
IMD Quintiles			
1^st^ Quintile (least deprived)	13,470 (17.5)	5,801 (15.1)	7,669 (19.9)
2^nd^ Quintile	16,788 (21.8)	7,814 (20.3)	8,974 (23.3)
3^rd^ Quintile	15,223 (19.8)	7,446 (19.3)	7,777 (20.2)
4^th^ Quintile	16,291 (21.1)	8,794 (22.8)	7,497 (19.5)
5^th^ Quintile (most deprived)	15,230 (19.8)	8,656 (22.5)	6,574 (17.1)

COPD = chronic obstructive pulmonary disease. CPRD = Clinical Practice Research Datalink general practice database.

**Table 2:** Unadjusted and adjusted associations between risk factors and diagnosis of COPD: Odds ratio (OR), 95% confidence interval (95% CI).

	Unadjusted OR (95% CI) *N* = 77,002	Adjusted^1^ OR (95% CI) *N* = 77,002
Smoking status		
Never smoker	1	1
Ever smoker	7.01 (6.70–7.33)	7.21 (5.88–7.57)
Previous asthma prior to diagnosis of COPD		
No	1	1
Yes	4.52 (4.31–4.74)	5.04 (4.77–5.33)
Index of Multiple Deprivation Quintiles		
1^st^ Quintile (least deprived)	1	1
2^nd^ Quintile	1.34 (1.27–1.41)	1.23 (1.16–1.31)
3^rd^ Quintile	1.64 (1.55–1.73)	1.47 (1.37–1.57)
4^th^ Quintile	2.20 (2.07–2.32)	1.81 (1.68–1.94)
5^th^ Quintile (most deprived)	2.90 (2.72–3.09)	2.17 (2.00–2.34)

COPD = chronic obstructive pulmonary disease. CPRD = Clinical Practice Research Datalink general practice database. IMD = Index of Multiple Deprivation.

**Table 3:** Validation of COPD prognostic scores derived from the PCCIU data using the CPRD data.

	Range of values for deciles of PI scores										Area under ROC curve (95% CI)
	1 (lowest)	2	3	4	5	6	7	8	9	10 (highest)	
Prognostic scores											
Males	0.00–0.00	0.61–1.41	1.42–1.91	—	1.92–2.52	—	2.53–3.31	—	3.32–4.49	4.50–5.71	0.66 (0.65– 0.66)
Females	0.00–0.00	0.01–0.45	0.46–1.50	1.51–2.26	—	2.27–2.71	2.72–3.73	3.74–3.76	3.77–4.93	4.94–5.95	0.71 (0.70–0.71)

COPD = chronic obstructive pulmonary disease. PCCIU = Primary Care Clinical Informatics Unit general practice database. CPRD = Clinical Practice Research Datalink general practice database. ROC = receiver operating characteristics.

Some deciles did not have corresponding prognostic scores, an indication that the scores were not normally distributed.

**Table 4:** Prognostic scores derived from the CPRD data.

	Range of values for deciles of prognostic scores										Area under ROC curve (95% CI)
	1 (lowest)	2	3	4	5	6	7	8	9	10 (highest)	
Prognostic scores	0.00–.21	0.22–0.59	0.60–1.98	1.99–2.19	—	2.20–2.36	2.37–2.57	2.58–2.75	2.76–3.80	3.81–4.37	0.74 (0.73–0.74)

CPRD = Clinical Practice Research Datalink general practice database. ROC = receiver operating characteristic.

**Table 5:** Calculation of prediction risk scores for males and females derived from the PCCIU dataset.

**Prognostic scores for Males**
0.722590422761375000*AgeGroup2 + 1.354003015630730000*AgeGroup3 +
1.794461805033540000*AgeGroup3 + 2.268067060819270000*AgeGroup5 +
2.640068571991060000*AgeGroup6 + 3.462262041329010000* AgeGroup7 +
1.905731297921800000*Smoking + 0.307312890106045000*IMD2 +
0.468566344550995000*IMD3 + 0.647039365745038000*IMD4 +
0.926189176930742000*IMD5 + 1.214816131312250000*Asthma
**Prognostic scores for Females**
0.719505024838863000*AgeGroup2 + 1.311267194962520000*AgeGroup3 +
1.702959068049090000*AgeGroup4 + 2.098189831042430000*AgeGroup5
2.352907331580150000*AgeGroup6 + 3.248496488525190000*AgeGroup7
2.262261867746550000*Smoking + 0.223346323577682000*IMD2 +
0.498918486471617000*IMD3 + 0.666610932992558000*IMD4 +
0.948500789111445000*IMD5 + 1.025043519409850000*Asthma

Age categories: AgeGroup1 (35–39 yrs: reference); AgeGroup2 (40–44 yrs); AgeGroup3 (45–49 yrs); AgeGroup4 (50–54 yrs); AgeGroup5 (55–59 yrs); AgeGroup6 (60–64 yrs); AgeGroup7 (65 + yrs).

IMD categories: IMD1 (1^st^ Quintile, least deprived: reference); IMD2 (2^nd^ Quintile); IMD3 (3^rd^ Quintile); IMD4 (4^th^ Quintile); IMD5 (5^th^ Quintile, most deprived).
