# Supplementary material for: External validation of a COPD prediction model using population-based primary care data: a nested case-control study
Source: Sci Rep. 2017 Mar 17;7:44702. doi: 10.1038/srep44702 (PMC5356333; doi:10.1038/srep44702)
Supplement: Supplementary Information [file srep44702-s1.pdf]

External validation of a COPD prediction model using population-based primary care data: a nested case-control study

Bright I Nwaru,[1,2] Colin R Simpson,[1] Aziz Sheikh,[1,3] Daniel Kotz,[1,3,4*]

[1]Asthma UK Centre for Applied Research, Centre for Medical Informatics, Usher Institute of Population Health Sciences, The University of Edinburgh, UK

[2]School of Health Sciences, University of Tampere, Finland

[3]Department of Family Medicine, CAPHRI School for Public Health and Primary Care, Maastricht University Medical Centre, Maastricht, The Netherlands

[4]Institute of General Practice, Medical Faculty of the Heinrich-Heine-University Düsseldorf, Düsseldorf, Germany

Correspondence:
Prof. Dr. Daniel Kotz
Institute of General Practice
Medical Faculty of the Heinrich-Heine-University
Moorenstr. 5
40225 Düsseldorf, Germany
Email: daniel.kotz@med.uni-duesseldorf.de
Tel: 0049-211-81-16019
Web: www.daniel-kotz.de

Supplementary File 1:
External validation of a COPD prediction model using population-based primary care data: a nested case-control study

Read codes used to define smoking status

Read code and definition
1372: Trivial smoker - < 1 cig/day[$$$]
1374: Moderate smoker - 10-19 cigs/d[$$$]
1376: Very heavy smoker - 40+cigs/d[$$$]
137G: Trying to give up smoking[$$$]
137J: Cigar smoker[$$$]
137P: Cigarette smoker[$$$]
137R: Current smoker[$$$]
137X: Cigarette consumption[$$$]
137Z: Tobacco consumption NOS[$$$]
137b: Ready to stop smoking[$$$]
137d: Not interested in stopping smoking[$$$]
137f: Reason for restarting smoking[$$$]
137h: Minutes from waking to first tobacco consumption[$$$]
1373: Light smoker - 1-9 cigs/day[$$$]
1375: Heavy smoker - 20-39 cigs/day[$$$]
137C: Keeps trying to stop smoking[$$$]
137H: Pipe smoker[$$$]
137M: Rolls own cigarettes[$$$]
137Q: Smoking started[$$$]
137V: Smoking reduced[$$$]
137Y: Cigar consumption[$$$]
137a: Pipe tobacco consumption[$$$]
137c: Thinking about stopping smoking[$$$]
137e: Smoking restarted[$$$]
137g: Cigarette pack-years[$$$]
1377: Ex-trivial smoker (<1/day) [$$$]
1378: Ex-light smoker (1-9/day) [$$$]
1379: Ex-moderate smoker (10-19/day) [$$$]
137A: Ex-heavy smoker (20-39/day) [$$$]
137B: Ex-very heavy smoker (40+/day) [$$$]
137F: Ex-smoker - amount unknown[$$$]
137K: Stopped smoking[$$$]
137N: Ex pipe smoker[$$$]
137O: Ex cigar smoker[$$$]
137S: Ex-smoker[$$$]
137T: Date ceased smoking[$$$]
137j: Ex-cigarette smoker[$$$]
137P.11: Smoker***
137K000: Recently stopped smoking***
ZV4K000: [V]Tobacco use***
9OO1.00: Attends stop smoking monitor***
1371: Never smoked tobacco[$$$]
137L: Current non-smoker[$$$]
137E.00: Tobacco consumption unknown***

[$$$]Codes found in both the Primary Care Clinical Informatics Unit and Clinical Practice Research Datalink databases
***Codes found only in Clinical Practice Research Datalink database

Read codes used to define prior asthma

Read code and definition
H33..00: Asthma***
H333.00: Acute exacerbation of asthma[$$$]
H33z100: Asthma attack[$$$]
H33z011: Severe asthma attack***
H330.12: Childhood asthma*
H33..11: Bronchial asthma***
H330.11: Allergic asthma***
H331.11: Late onset asthma***
H33z.00: Asthma unspecified[$$$]
H33zz11: Exercise induced asthma***
H33z000: Status asthmaticus NOS[$$$]
H331.00: Intrinsic asthma[$$$]
H330011: Hay fever with asthma***
H330111: Extrinsic asthma with asthma attack***
H330.00: Extrinsic (atopic) asthma[$$$]
H330.14: Pollen asthma***
H33z111: Asthma attack NOS***
H33z200: Late-onset asthma[$$$]
H330000: Extrinsic asthma without status asthmaticus[$$$]
H330.13: Hay fever with asthma***
H33zz00: Asthma NOS[$$$]
H331111: Intrinsic asthma with asthma attack***
H33zz12: Allergic asthma NEC***
H332.00: Mixed asthma[$$$]
H330100: Extrinsic asthma with status asthmaticus[$$$]
H331000: Intrinsic asthma without status asthmaticus[$$$]
H334.00: Brittle asthma***
H331z00: Intrinsic asthma NOS[$$$]
H330z00: Extrinsic asthma NOS[$$$]
H331100: Intrinsic asthma with status asthmaticus[$$$]

[$$$]Codes found in both the Primary Care Clinical Informatics Unit and Clinical Practice Research Datalink databases

***Codes found only in Clinical Practice Research Datalink database

Supplementary File 2:
SYNTAXES FOR PREPARING THE DATA SETS

**Large data files - e.g Clinical data set - came in smaller barges. These data sets where then first merged in preparation for further analyses

```
*******************************************************************************
**FOR THE CURRENT ANALYSIS, THE RELEVANT DATA SETS INCLUDED:

*1. Matched data set: This data set contains information on the matching of COPD cases
*and controls and the various matching variables, *including gender, practice,
*date of birth, age, and date of event*

*2. Clinical data set: This contains medical history events - i.e. all the medical
*history data entered on the GP *system, including symptoms, signs and diagnoses.
*This can be used to identify any clinical diagnoses, and deaths. *Patients may
*have more than one row of data. The data is coded using Read codes, which allow
*linkage of codes to the medical terms provided.*

*3. SES data set: This data set contains information on participants' classification
*into the quintiles of Index of Multiple Deprivation*

*4. Mortality data set: This data set contains information on the death status of
*participants as defined by the Office of National Statistics (ONS)*

*******************************************************************************

*Set working directory*

cd "F:\Bright Kotz COPD"

**Preparing COPD Case-Control Matched Data Set**

use "C:\CPRD COPD data\Bright\Matching_results_final.dta", clear

sort case_patid, stable

gen casecont1=_n if case_patid~=.

gen casecont2=_n if control_patid~=.

rename case_patid patid1

rename control_patid patid2

rename case_pracid pracid1

rename control_pracid pracid2

rename case_gender gender1

rename control_gender gender2

rename case_yob yob1

rename control_yob yob2

drop if patid2==0

gen commonID=_n
```

```
save "C:\CPRD COPD data\Bright\Analysis Files\Matched_files_renamed", replace
**Deriving case index date variable**
gen case_indexday = regexs(0) if regexm(case_index, "^[0-9]+")
gen case_indexmonth = regexs(0) if regexm(case_index, "[a-zA-Z]+")
gen case_indexyear = regexs(0) if regexm(case_index, "[0-9]*$")
replace case_indexyear = "20"+regexs(0) if regexm(case_indexyear, "^[0-1][0-9]$")
replace case_indexyear = "19"+regexs(0) if regexm(case_indexyear, "^[2-9][0-9]$")
gen case_index2 = case_indexday+case_indexmonth+case_indexyear
gen case_index3 = date(case_index2, "DMY")
format case_index3 %td
drop case_index case_index2 case_indexday case_indexmonth case_indexyear
rename case_index3 case_index
save, replace

**Reshaping data from wide to long format**
use "C:\CPRD COPD data\Bright\Analysis Files\Matched_files_renamed", clear
reshape long patid pracid gender yob, i(commonID) j(casecont)
drop casecont1 casecont2
recode casecont (2=0)
gen case_index2=case_index
gen control_regstart2=control_regstart if casecont==0
gen control_regend2=control_regend if casecont==0
drop case_index control_regstart control_regend
rename control_regstart2 control_regstart
rename control_regend2 control_regend
label define casecont1 0 "control" 1 "case"
label values casecont casecont1
label variable casecont "COPD cases and controls"
save "C:\CPRD COPD data\Bright\Analysis Files\Matched_files_renamed_long", replace
*********************************************************************************

**Merging Clinical data sets - data came in 4 different data sets (PET_Clinical001,
```

PET_Clinical002, PET_Clinical003, PET_Clinical004)due to its size

```
use "F:\Bright Kotz COPD\PET_Clinical001.dta", clear

append using PET_Clinical002

append using PET_Clinical003

append using PET_Clinical004

save "F:\Bright Kotz COPD\PET_Clinical_ALL.dta"

********************************************************************************

**Generating smoking codes - from Clinical data set**

use "C:\CPRD COPD data\Bright\PET_Clinical_ALL.dta"

sort patid, stable

generate smokingcprd=9
foreach x in medcode {
	recode smokingcprd (9=1) if (`x'==12951 | `x'==12952 | `x'==12954 | `x'==12953 | `x'==12955
| `x'==12956 | `x'==12957 | `x'==12958 | `x'==12959 | `x'==12960 | `x'==12961 ///
	| `x'==12963 |`x'==12964 | `x'==12965 | `x'==12966 | `x'==12967 | `x'==1878 | `x'==12959 |
`x'==12240 | `x'==12943 | `x'==12944 | `x'==12945 | `x'==12946 | `x'==12947 | `x'==93 ///
	| `x'==10558 | `x'==31114 | `x'==46321 | `x'==30762 | `x'==62686 | `x'==1823 | `x'==3568 |
`x'==30423 | `x'==46300 | `x'==776 | `x'==99838 | `x'==26470 | `x'==19488 | `x'==90 ///
	| `x'==12878 | `x'==97210)
	recode smokingcprd (9=9) if (`x'==33 | `x'==60)
	recode smokingcprd (9=2) if (`x'==12962)
	}
**Label variables/values
label variable smokingcprd "Smoking status CPRD 1"
label define smoke 9 "never smoker" 1 "ever smoker" 2 "smoking unknown"
label values smokingcprd smoke

by patid: generate smokcprdseq1=sum(smokingcprd) if smokingcprd==1
by patid: generate smokcprdseq2=sum(smokingcprd) if smokingcprd==2
by patid: generate smokcprdseq3=sum(smokingcprd) if smokingcprd==9
generate smokingcprd2=.
recode smokingcprd2 (.=1) if smokcprdseq1==1
recode smokingcprd2 (.=2) if smokcprdseq2==2
label variable smokingcprd2 "Smoking status CPRD2 for analysis"
label define smoke2 1 "ever smoker" 2 "smoking unknown"
label values smokingcprd2 smoke2

save "C:\CPRD COPD data\Bright\PET_Clinical_ALL2.dta", replace

keep if smokingcprd2~=.

save "C:\CPRD COPD data\Bright\smokingcprd2.dta", replace

duplicates list patid

duplicates tag patid, gen(dupli)

gen smokingdupli=1 if dupli==1 & smokingcprd2==1

replace smokingdupli=2 if dupli==1 & smokingcprd2==2

drop if smokingdupli==2
```

```
drop dupli smokingdupli

generate smokedate=eventdate

generate smokecprd=smokingcprd2

save, replace


*Keep smoking codes for forward merging*

keep smokedate smokecprd patid

save "C:\CPRD COPD data\Bright\Smokingdata.dta", replace


*****************************************************************************

**Generating asthma and non asthma cases - Clinical data set**

use "C:\CPRD COPD data\Bright\PET_Clinical_ALL.dta", clear

sort patid, stable


generate asthmacprd=9
foreach x in medcode {
	recode asthmacprd (9=1) if (`x'==78 | `x'==185 | `x'==232 | `x'==233 | `x'==1208 | `x'==1555 | `x'==2290 | `x'==3665 | `x'==4442 | `x'==4606 | `x'==4892 ///
	| `x'==5267 |`x'==5627 | `x'==6707 | `x'==7146 | `x'==7731 | `x'==8335 | `x'==12987 | `x'==14777 | `x'==15248 | `x'==16070 | `x'==18323 | `x'==21232 ///
	| `x'==25796 | `x'==27926 | `x'==29325 | `x'==40823 | `x'==45073 | `x'==45782 | `x'==58196)
	}
*Label variables/values*
label variable asthmacprd "Asthma status based on CPRD data"
label define asthma 9 "no asthma" 1 "asthma"
label values asthmacprd asthma

by patid: generate asthmacprdseq1=sum(asthmacprd) if asthmacprd==1
by patid: generate asthmacprdseq2=sum(asthmacprd) if asthmacprd==9
generate asthmacprd2=.
recode asthmacprd2 (.=1) if asthmacprdseq1==1
recode asthmacprd2 (.=.) if asthmacprdseq2==9
label variable asthmacprd2 "Asthma status based on CPRD data for analysis"

save "C:\CPRD COPD data\Bright\PET_Clinical_ALL5.dta", replace

keep if asthmacprd2~=.

save "C:\CPRD COPD data\Bright\asthmacprd2.dta", replace

duplicates list patid

*No duplicates found*

save, replace

gen eventdate2 = date(eventdate, "DMY")

format eventdate2 %td
```

```
generate asthmadate=eventdate2

format asthmdate %td

save, replace

*Keep asthma codes and other relevant variables for forward merging*

keep asthmadate asthmacprd patid

save "C:\CPRD COPD data\Bright\Asthmadata.dta", replace

*****************************************************************************

**Creating SES variables for cases and controls**

use "C:\CPRD COPD data\Bright\SES_IMD_Data_10_084_NEW.dta", clear

generate ses=quintile

keep ses patid

save "C:\CPRD COPD data\Bright\SES_IMD_Data_10_084_NEW2.dta", replace

*****************************************************************************

**Creating mortality variable**

use "C:\CPRD COPD data\Bright\ONS_Mortality_Data_10_084_NEW.dta", clear

gen dod2 = date(dod, "DMY")

format dod2 %td

generate death=1 if dod2~=.

recode death (.=0)

keep dod2 patid death

save "C:\CPRD COPD data\Bright\ONS_Mortality_Data_10_084_NEW2.dta", replace

*****************************************************************************

**Generating physical activity variables**

use "C:\CPRD COPD data\Bright\PET_Clinical_ALL.dta", clear
sort patid, stable

generate GPPAQ=9
foreach x in medcode {
	recode GPPAQ (9=1) if (`x'==103188)
	}
**Label variables/values
label variable GPPAQ "General practice physical activity questionnaire"
label define answered 9 "not answered" 1 "answered"
label values GPPAQ answered
```

```
by patid: generate GPPAQseq1=sum(GPPAQ) if GPPAQ==1
by patid: generate GPPAQseq2=sum(GPPAQ) if GPPAQ==9
generate GPPAQ_2=.
recode GPPAQ_2 (.=1) if GPPAQseq1==1
recode GPPAQ_2 (.=.) if GPPAQseq2==9
label variable GPPAQ_2 "General practice physical activity questionnaire for analysis"

save "C:\CPRD COPD data\Bright\GPPAQ_2.dta", replace

keep if GPPAQ_2~=.

save "C:\CPRD COPD data\Bright\GPPAQ_2.dta", replace

duplicates list patid


**Generating moderately active physical activity variables**

use "C:\CPRD COPD data\Bright\PET_Clinical_ALL.dta", clear
sort patid, stable

generate GPPAQ1=9
foreach x in medcode {
	recode GPPAQ1 (9=1) if (`x'==95900)
	}
**Label variables/values
label variable GPPAQ1 "Moderately active physical activity"

label values GPPAQ1 answered

by patid: generate GPPAQ1seq1=sum(GPPAQ1) if GPPAQ1==1
by patid: generate GPPAQ1seq2=sum(GPPAQ1) if GPPAQ1==9
generate GPPAQ1_2=.
recode GPPAQ1_2 (.=1) if GPPAQ1seq1==1
recode GPPAQ1_2 (.=.) if GPPAQ1seq2==9
label variable GPPAQ1_2 "Moderately active physical activity for analysis"

save "C:\CPRD COPD data\Bright\GPPAQ1_2.dta", replace

keep if GPPAQ1_2~=.

save "C:\CPRD COPD data\Bright\GPPAQ1_2.dta", replace

duplicates list patid


**Generating inactive physical activity variables**

use "C:\CPRD COPD data\Bright\PET_Clinical_ALL.dta", clear
sort patid, stable

generate GPPAQ2=9
foreach x in medcode {
	recode GPPAQ2 (9=1) if (`x'==96213)
	}
**Label variables/values
label variable GPPAQ2 "Inactive physical activity"

label values GPPAQ2 answered

by patid: generate GPPAQ2seq1=sum(GPPAQ2) if GPPAQ2==1
```

```
by patid: generate GPPAQ2seq2=sum(GPPAQ2) if GPPAQ2==9
generate GPPAQ2_2=.
recode GPPAQ2_2 (.=1) if GPPAQ2seq1==1
recode GPPAQ2_2 (.=.) if GPPAQ2seq2==9
label variable GPPAQ2_2 "Inactive physical activity for analysis"

save "C:\CPRD COPD data\Bright\GPPAQ2_2.dta", replace

keep if GPPAQ2_2~=.

save "C:\CPRD COPD data\Bright\GPPAQ2_2.dta", replace

duplicates list patid


**Generating active physical activity variables**

use "C:\CPRD COPD data\Bright\PET_Clinical_ALL.dta", clear
sort patid, stable

generate GPPAQ3=9
foreach x in medcode {
	recode GPPAQ3 (9=1) if (`x'==96647)
	}
**Label variables/values
label variable GPPAQ3 "Active physical activity"

label values GPPAQ3 answered

by patid: generate GPPAQ3seq1=sum(GPPAQ3) if GPPAQ3==1
by patid: generate GPPAQ3seq2=sum(GPPAQ3) if GPPAQ3==9
generate GPPAQ3_2=.
recode GPPAQ3_2 (.=1) if GPPAQ3seq1==1
recode GPPAQ3_2 (.=.) if GPPAQ3seq2==9
label variable GPPAQ3_2 "Active physical activity for analysis"

save "C:\CPRD COPD data\Bright\GPPAQ3_2.dta", replace

keep if GPPAQ3_2~=.

save "C:\CPRD COPD data\Bright\GPPAQ3_2.dta", replace

duplicates list patid


**Generating moderately inactive physical activity variables**

use "C:\CPRD COPD data\Bright\PET_Clinical_ALL.dta", clear
sort patid, stable

generate GPPAQ4=9
foreach x in medcode {
	recode GPPAQ4 (9=1) if (`x'==97179)
	}
**Label variables/values
label variable GPPAQ4 "Moderately inactive physical activity"

label values GPPAQ4 answered

by patid: generate GPPAQ4seq1=sum(GPPAQ4) if GPPAQ4==1
by patid: generate GPPAQ4seq2=sum(GPPAQ4) if GPPAQ4==9
generate GPPAQ4_2=.
```

```
recode GPPAQ4_2 (.=1) if GPPAQ4seq1==1
recode GPPAQ4_2 (.=.) if GPPAQ4seq2==9
label variable GPPAQ4_2 "Moderately inactive physical activity for analysis"

save "C:\CPRD COPD data\Bright\GPPAQ4_2.dta", replace

keep if GPPAQ4_2~=.

save "C:\CPRD COPD data\Bright\GPPAQ4_2.dta", replace

duplicates list patid


**Merging all physical activity data into one dataset**

*Merging GPPAQ_2 and GPPAQ1_2

use "C:\CPRD COPD data\Bright\GPPAQ1_2.dta", clear
sort patid, stable
save, replace

use "C:\CPRD COPD data\Bright\GPPAQ_2.dta", clear
sort patid, stable
save, replace

merge 1:1 patid using "C:\CPRD COPD data\Bright\GPPAQ1_2.dta"

save "C:\CPRD COPD data\Bright\GPPAQ.dta", replace

*Drop merging variable*

drop _merge

save, replace


*Merging GPPAQ and GPPAQ2_2

use "C:\CPRD COPD data\Bright\GPPAQ2_2.dta", clear
sort patid, stable
save, replace

use "C:\CPRD COPD data\Bright\GPPAQ.dta", clear
sort patid, stable
save, replace

merge 1:1 patid using "C:\CPRD COPD data\Bright\GPPAQ2_2.dta"

save "C:\CPRD COPD data\Bright\GPPAQ.dta", replace

*Drop merging variable*

drop _merge

save, replace


*Merging GPPAQ and GPPAQ3_2

use "C:\CPRD COPD data\Bright\GPPAQ3_2.dta", clear
sort patid, stable
save, replace
```

```
use "C:\CPRD COPD data\Bright\GPPAQ.dta", clear
sort patid, stable
save, replace

merge 1:1 patid using "C:\CPRD COPD data\Bright\GPPAQ3_2.dta"

save "C:\CPRD COPD data\Bright\GPPAQ.dta", replace

*Drop merging variable*

drop _merge

save, replace


*Merging GPPAQ and GPPAQ4_2

use "C:\CPRD COPD data\Bright\GPPAQ4_2.dta", clear
sort patid, stable
save, replace

use "C:\CPRD COPD data\Bright\GPPAQ.dta", clear
sort patid, stable
save, replace

merge 1:1 patid using "C:\CPRD COPD data\Bright\GPPAQ4_2.dta"

save "C:\CPRD COPD data\Bright\GPPAQ.dta", replace

*Drop merging variable*

drop _merge

save, replace


**Generate one variable for all physical activity variables**

gen GPPAQ_all=.
replace GPPAQ_all=1 if GPPAQ1_2==1
replace GPPAQ_all=2 if GPPAQ2_2==1
replace GPPAQ_all=3 if GPPAQ3_2==1
replace GPPAQ_all=4 if GPPAQ4_2==1

label define GPPAQall 1 "moderately_active" 2 "inactive" 3 "active" 4 "moderately_inactive"

label values GPPAQ_all GPPAQall

label variable GPPAQ_all "Physical activity status"

drop GPPAQ-GPPAQ4_2

save "C:\CPRD COPD data\Bright\GPPAQ_ALL.dta", replace


*******************************************************************************************

***MERGING ALL FILES***
**Files to merge**
*Matched_files_renamed_long (base file): "C:\CPRD COPD data\Bright\Analysis
Files\Matched_files_renamed_long""*
```

```
*Asthma data: "C:\CPRD COPD data\Bright\Asthmadata.dta"
*Smoking data: "C:\CPRD COPD data\Bright\Smokingdata.dta"
*SES data: "C:\CPRD COPD data\Bright\SES_IMD_Data_10_084_NEW2.dta"
*Mortality data: "C:\CPRD COPD data\Bright\ONS_Mortality_Data_10_084_NEW2.dta"

********************************************************************************

**Merging Asthma data with Matching-final data**

use "C:\CPRD COPD data\Bright\Asthmadata.dta", clear
sort patid, stable
save, replace

use "C:\CPRD COPD data\Bright\Analysis Files\Matched_files_renamed_long", clear
sort patid, stable
save, replace

merge 1:1 patid using "C:\CPRD COPD data\Bright\Asthmadata.dta"

save "C:\CPRD COPD data\Bright\Analysis Files\Matched_files_renamed_longAsthma.dta", replace

*Drop merging variable*

drop _merge

save, replace


*********************************************************************************


**Merging Smoking and Matched_files_renamed_longAsthma data sets**

use "C:\CPRD COPD data\Bright\Smokingdata.dta", clear
sort patid, stable
save, replace

use "C:\CPRD COPD data\Bright\Analysis Files\Matched_files_renamed_longAsthma.dta", clear
sort patid, stable
save, replace

merge 1:1 patid using "C:\CPRD COPD data\Bright\Smokingdata.dta"

save "C:\CPRD COPD data\Bright\Analysis Files\Matched_files_renamed_longAsthmaSmoking.dta",
replace

*Drop merging variable*

drop _merge

save, replace


*********************************************************************************

**Merging SES and MatchingAsthmaSmoking data sets**

use "C:\CPRD COPD data\Bright\SES_IMD_Data_10_084_NEW2.dta", clear
sort patid, stable
save, replace
```

```
use "C:\CPRD COPD data\Bright\Analysis Files\Matched_files_renamed_longAsthmaSmoking.dta", clear
sort patid, stable
save, replace

merge 1:1 patid using "C:\CPRD COPD data\Bright\SES_IMD_Data_10_084_NEW2.dta"

save "C:\CPRD COPD data\Bright\Analysis Files\Matched_files_renamed_longAsthmaSmokingSES.dta", replace

*Drop merging variable*

drop _merge

save, replace


*********************************************************************************

**Merging MatchingAsthmaSmokingSES and Mortality data sets**

use "C:\CPRD COPD data\Bright\ONS_Mortality_Data_10_084_NEW2.dta", clear
sort patid, stable
save, replace

use "C:\CPRD COPD data\Bright\Analysis Files\Matched_files_renamed_longAsthmaSmokingSES.dta", clear
sort patid, stable
save, replace

merge 1:1 patid using "C:\CPRD COPD data\Bright\ONS_Mortality_Data_10_084_NEW2.dta"

save "C:\CPRD COPD data\Bright\Analysis Files\Matched_files_renamed_longAsthmaSmokingSESMortality.dta", replace

*Drop unmatched records*

drop _merge

save, replace


*********************************************************************************

**Merging MatchingAsthmaSmokingSES and Physical Activity data sets**

use "C:\CPRD COPD data\Bright\GPPAQ_ALL.dta", clear
sort patid, stable
save, replace

use "C:\CPRD COPD data\Bright\Analysis Files\Matched_files_renamed_longAsthmaSmokingSESMortality.dta", clear
sort patid, stable
save, replace

merge 1:1 patid using "C:\CPRD COPD data\Bright\GPPAQ_ALL.dta"

save "C:\CPRD COPD data\Bright\Analysis Files\Matched_files_renamed_longAsthmaSmokingSESMortalityGPPAQ_ALL.dta", replace

*Drop unmatched records*
```

```
drop _merge

save, replace

********************************************************************************

***ENSURING THAT ASTHMA CASES CAME BEFORE COPD DIAGNOSIS***

*Calculate differeces between date of asthma and date of copd*

*Generate new working date variables and format the date variables to Stata days first*

use "C:\CPRD COPD data\Bright\Analysis
Files\Matched_files_renamed_longAsthmaSmokingSESMortalityGPPAQ_ALL.dta", clear

sort commonID, stable

save, replace

gen smokedate2 = date(smokedate, "DMY")

format smokedate2 %td

format case_index2 %td

format asthmadate %td

*Previous asthma before diagnosis of COPD*

generate asthma_copd=case_index2-asthmadate if asthmacprd==1

*Previous asthma <1 day before COPD*

gen asthmacprd_1day=asthmacprd

replace asthmacprd_1day=. if asthma_copd<1

label variable asthmacprd_1day "Asthma at least 1 day b4 COPD"

recode asthmacprd_1day smokecprd death GPPAQ_all(.=0)

recode smokecprd (2=.)

label variable smokecprd "Smoking status"

label variable death "Death status"

label define ses1 0 "1st_IMD_Quintile" 1 "2nd_IMD_Quintile" 2 "3rd_IMD_Quintile" 3
"4th_IMD_Quintile" 4 "5th_IMD_Quintile"

label values ses ses1

label variable ses "IMD Quintiles"

label define sex 1 "male" 2 "female"

label values gender sex

keep if smokecprd~=.

label define GPPAQall2 0 "No_GPPAG" 1 "moderately_active" 2 "inactive" 3 "active" 4
"moderately_inactive"
```

```
label values GPPAQ_all GPPAQall2

label define smokeasthma 0 "No" 1 "Yes

label values asthmacprd_1day smokecprd smokeasthma

keep if ses~=.

**Generate an indicator variable for each gender**

gen males=1 if gender==1
gen females=1 if gender==2
recode males females (.=0)

gen ses_2=2 if ses==2
gen ses_3=3 if ses==3
gen ses_4=4 if ses==4
gen ses_5=5 if ses==5

recode ses_2 ses_3 ses_4 ses_5 (.=0)

save, replace

********************************************************************************
```

Supplementary File 3:
DESCRIPTIVE ANALYSES

tab smokecprd casecont, missing column

tab asthmacprd_1day casecont, missing column

tab ses casecont, missing column

****

UNADJUSTED CONDITIONAL LOGISTIC REGRESSION MODELS

Smoking and COPD

clogit casecont smokecprd, group(commonID) or

Previous asthma and COPD

clogit casecont asthmacprd_1day, group(commonID) or

SES-IMD and COPD

clogit casecont i.ses, group(commonID) or

testparm i(1/4).ses

****

ADJUSTED MODELS

Smoking and COPD

clogit casecont asthmacprd_1day i.ses smokecprd, group(commonID) or

Previous asthma and COPD

clogit casecont smokecprd i.ses asthmacprd_1day, group(commonID) or

SES - IMD

clogit casecont asthmacprd_1day smokecprd i.ses, group(commonID) or

testparm i(1/4).ses

****

Calculation of Prognostic Index (PI) Scores

1. Applying PI scores from previous study to males and females separately

gen PImales = 1.905731297921800000*smokecprd + 0.307312890106045000*ses_2 + 0.468566344550995000*ses_3 + 0.647039365745038000*ses_4 + 0.926189176930742000*ses_5 + 1.214816131312250000*asthmapccui_1day if males==1

gen PIfemales = 2.262261867746550000*smokecprd + 0.223346323577682000*ses_2 + 0.498918486471617000*ses_3 + 0.666610932992558000*ses_4 + 0.948500789111445000*ses_5 + 1.025043519409850000*asthmapccui_1day if females==1

*Deciles of above PI scores**

xtile PImales_xtile = PImales if males==1, nq(10)

xtile PIfemales_xtile = PIfemales if females==1, nq(10)

save, replace

Summary results*

summarize PImales, detail

sort PImales_xtile

by PImales_xtile: summarize PImales

summarize PIfemales, detail

sort PIfemales_xtile

by PIfemales_xtile: summarize PIfemales

2. Derive coefficients and calculate PI scores

First run the model

clogit casecont smokecprd i.ses asthmacprd_1day, group(commonID)

Generate the PI scores

gen PICPRD = _b[smokecprd]*smokecprd + _b[asthmacprd_1day]*asthmacprd_1day + _b[1.ses]*1.ses + _b[2.ses]*2.ses + _b[3.ses]*3.ses + _b[4.ses]*4.ses

Deciles of above PI scores

xtile PICPRD_xtile = PICPRD, nq(10)

save, replace

Summary results*

summarize PICPRD, detail

sort PICPRD_xtile

by PICPRD_xtile: summarize PICPRD

****

ROC CURVES

Validation of PI scores for Males

roctab casecont PImales, table graph summary

roctab casecont PImales, detail

roctab casecont PImales, bamber

Validation of PI scores for Females

roctab casecont PIfemales, table graph summary

roctab casecont PIfemales, detail

roctab casecont PIfemales, bamber

Deriving scores based on CPRD data

roctab casecont PICPRD, table graph summary

roctab casecont PICPRD, detail

roctab casecont PICPRD, bamber

Supplementary File 4: Sensitivity and specificity values for the various cut points on the prognostic index

	MALES			
**Cut-point on PI***	**Sensitivity**	**Specificity**	**Correctly classified**	**Negative likelihood ratio**
>= 0	100.00%	0.00%	50.01%	1,00
>= .31	98.41%	9.34%	53.89%	1,09
>= .47	96.29%	19.03%	57.67%	1,19
>= .65	94.30%	27.02%	60.67%	1,29
>= .93	92.34%	34.18%	63.27%	1,40
>= 1.21	90.66%	39.39%	65.03%	1,50
>= 1.52	90.43%	39.54%	64.99%	1,50
>= 1.68	90.22%	39.65%	64.94%	1,49
>= 1.86	89.99%	39.76%	64.88%	1,49
>= 1.90	89.83%	39.84%	64.84%	1,49
>= 2.14	76.24%	51.20%	63.72%	1,56
>= 2.21	76.08%	51.28%	63.68%	1,56
>= 2.37	58.47%	64.78%	61.62%	1,66
>= 2.55	42.26%	76.53%	59.39%	1,80
>= 2.83	23.41%	88.49%	55.94%	2,03
>= 3.12	5.35%	99.09%	52.21%	5,85
>= 3.43	4.56%	99.28%	51.91%	6,34
>= 3.59	3.37%	99.47%	51.41%	6,33
>= 3.77	2.36%	99.72%	51.03%	8,32
>= 4.05	1.10%	99.87%	50.47%	8,29
> 4.05	0.00%	100.00%	49.99%	

*PI=Prognostic index

	FEMALES		
**Positive likelihood ratio**	**Cut-point on PI***	**Sensitivity**	**Specificity**
	>= 0	100.00%	0.00%
0,17	>= .22	97.30%	12.02%
0,19	>= .49	93.92%	25.94%
0,21	>= .67	90.97%	37.66%
0,22	>= .95	87.97%	47.90%
0,24	>= 1.02	85.23%	56.31%
0,24	>= 1.25	84.77%	56.52%
0,25	>= 1.52	84.24%	56.89%
0,25	>= 1.69	83.64%	57.22%
0,26	>= 1.97	83.08%	57.50%
0,46	>= 2.26	82.67%	57.74%
0,47	>= 2.48	73.05%	64.01%
0,64	>= 2.76	59.07%	72.56%
0,75	>= 2.93	45.06%	80.55%
0,87	>= 3.21	27.06%	89.40%
0,96	>= 3.29	8.28%	98.72%
0,96	>= 3.51	7.31%	98.89%
0,97	>= 3.79	5.84%	99.17%
0,98	>= 3.95	4.20%	99.45%
0,99	>= 4.23	2.23%	99.69%
1,00	> 4.23	0.00%	100.00%

Correctly classified	Negative likelihood ratio	Positive likelihood ratio
50.01%	1,00	
54.67%	1,11	0,22
59.94%	1,27	0,23
64.32%	1,46	0,24
67.94%	1,69	0,25
70.77%	1,95	0,26
70.65%	1,95	0,27
70.56%	1,95	0,28
70.43%	1,96	0,29
70.30%	1,96	0,29
70.21%	1,96	0,30
68.53%	2,03	0,42
65.81%	2,15	0,56
62.80%	2,32	0,68
58.22%	2,55	0,82
53.49%	6,47	0,93
53.09%	6,57	0,94
52.50%	7,08	0,95
51.81%	7,59	0,96
50.95%	7,19	0,98
49.99%		1,00

Supplementary File 5:

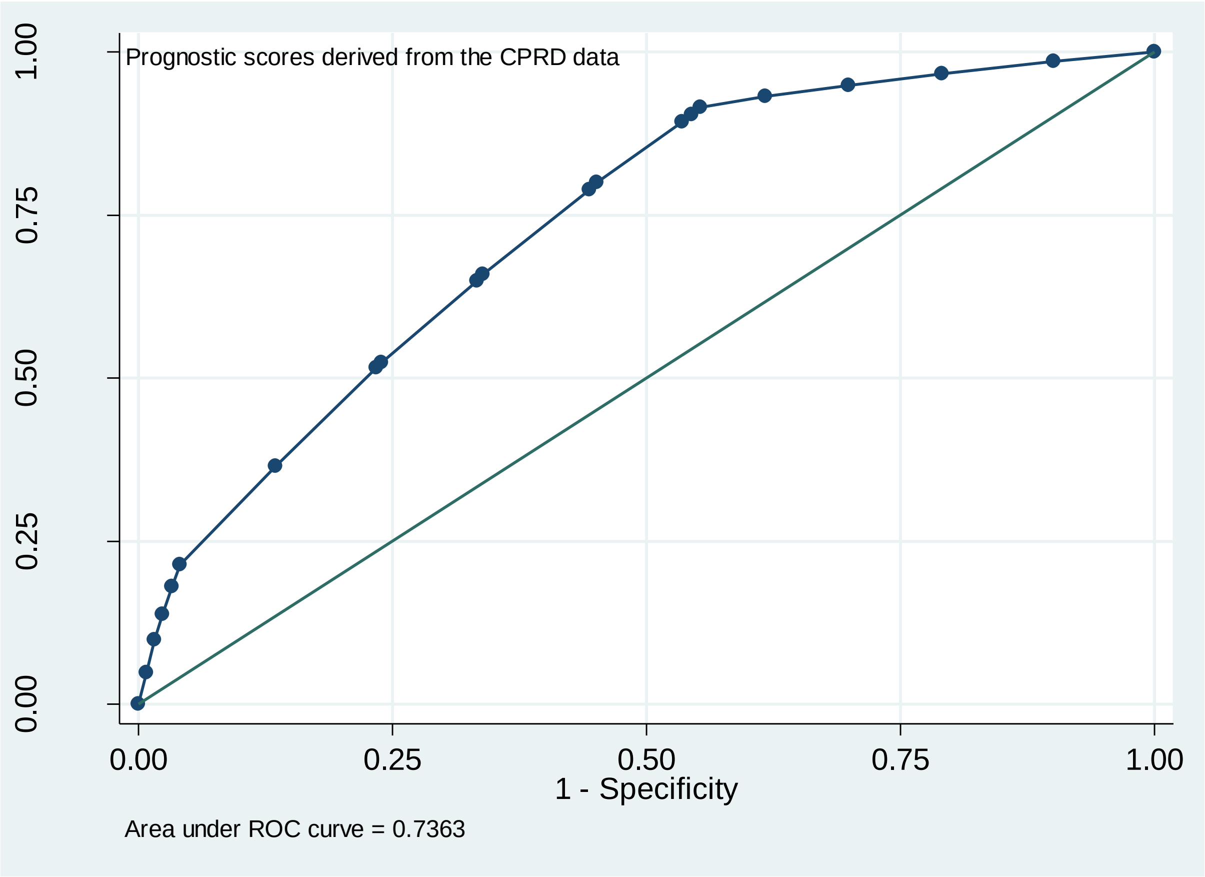

Figure S1 ROC curves for the prognostic scores derived using solely the CPRD data

Supplementary File 5:

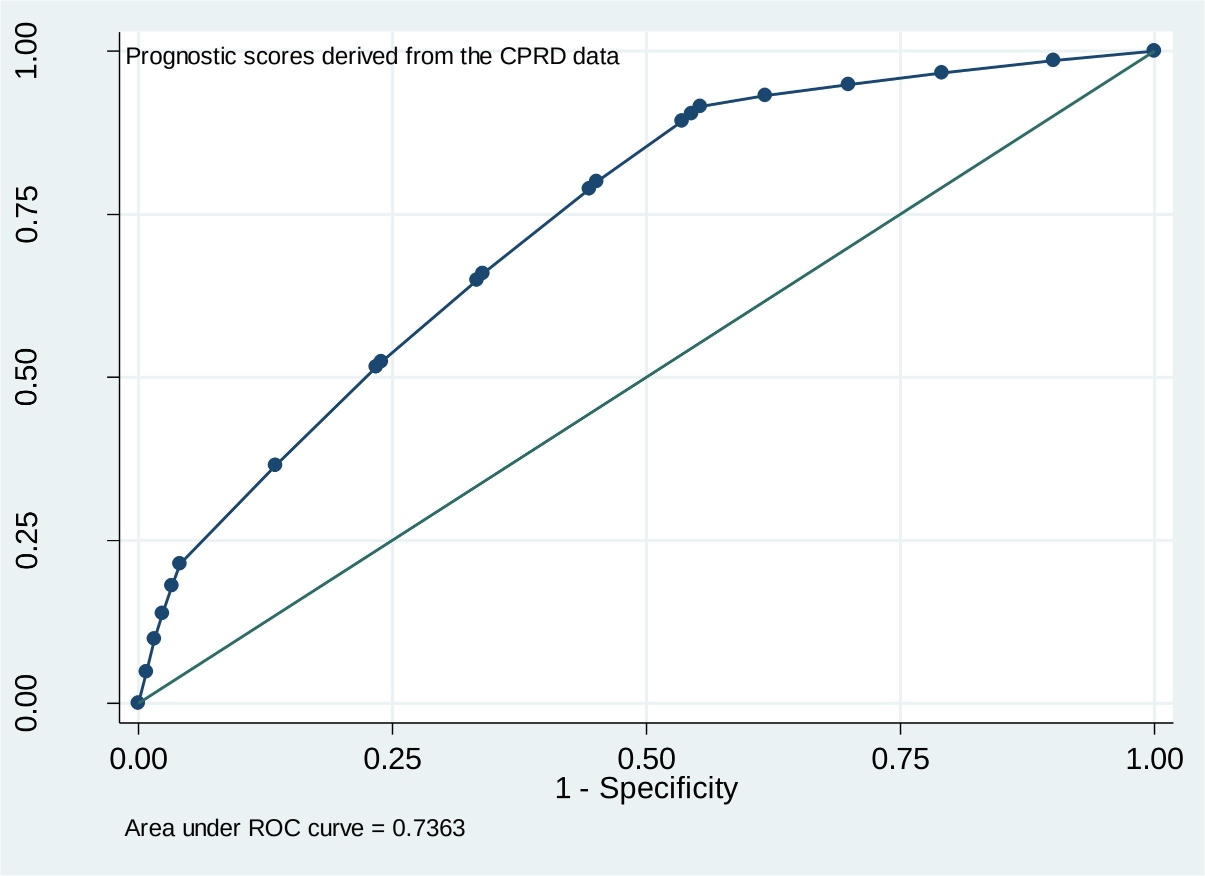

Figure S1 ROC curves for the prognostic scores derived using solely the CPRD data
